# The Rts1 Regulatory Subunit of Protein Phosphatase 2A Is Required for Control of G1 Cyclin Transcription and Nutrient Modulation of Cell Size

**DOI:** 10.1371/journal.pgen.1000727

**Published:** 2009-11-13

**Authors:** Karen Artiles, Stephanie Anastasia, Derek McCusker, Douglas R. Kellogg

**Affiliations:** Department of Molecular, Cell and Developmental Biology, University of California Santa Cruz, Santa Cruz, California, United States of America; National Institute of Diabetes and Digestive and Kidney Diseases, United States of America

## Abstract

The key molecular event that marks entry into the cell cycle is transcription of G1 cyclins, which bind and activate cyclin-dependent kinases. In yeast cells, initiation of G1 cyclin transcription is linked to achievement of a critical cell size, which contributes to cell-size homeostasis. The critical cell size is modulated by nutrients, such that cells growing in poor nutrients are smaller than cells growing in rich nutrients. Nutrient modulation of cell size does not work through known critical regulators of G1 cyclin transcription and is therefore thought to work through a distinct pathway. Here, we report that Rts1, a highly conserved regulatory subunit of protein phosphatase 2A (PP2A), is required for normal control of G1 cyclin transcription. Loss of Rts1 caused delayed initiation of bud growth and delayed and reduced accumulation of G1 cyclins. Expression of the G1 cyclin *CLN2* from an inducible promoter rescued the delayed bud growth in *rts1*Δ cells, indicating that Rts1 acts at the level of transcription. Moreover, loss of Rts1 caused altered regulation of Swi6, a key component of the SBF transcription factor that controls G1 cyclin transcription. Epistasis analysis revealed that Rts1 does not work solely through several known critical upstream regulators of G1 cyclin transcription. Cells lacking Rts1 failed to undergo nutrient modulation of cell size. Together, these observations demonstrate that Rts1 is a key player in pathways that link nutrient availability, cell size, and G1 cyclin transcription. Since Rts1 is highly conserved, it may function in similar pathways in vertebrates.

## Author Summary

A critical point in the cell cycle occurs in G1 phase, when cells must decide whether to enter a new round of cell division. At this time, cells assess nutrient availability to ensure that they have sufficient resources to complete cell growth and division. Vertebrate cells also assess growth factors that control cell growth and determine when and where cell division occurs in the context of a multi-cellular organism. A cell-size checkpoint acts during G1 to delay entry into the cell cycle if the cell is below a critical size. When the appropriate signals have been received, cells commit to entry into the cell cycle by initiating transcription of G1 cyclins. The mechanisms that integrate external signals, cell growth, cell size, and entry into the cell cycle are poorly understood and represent a fundamental unsolved problem in cell biology. We discovered that a specific form of protein phosphatase 2A (PP2A^Rts1^) functions in the pathways that integrate nutrient availability, cell size, and entry into the cell cycle. PP2A^Rts1^ is highly conserved and may therefore carry out similar functions in all eukaryotic cells.

## Introduction

Entry into the cell cycle is initiated by G1 cyclins, which bind and activate cyclin-dependent kinases [1]. There are two cyclin-dependent kinases in budding yeast that function during G1, called Cdk1 and Pho85, which are activated by numerous different G1 cyclins [1]. Cdk1 is activated by the cyclins Cln1, Cln2, and Cln3, while Pho85 is activated by Pcl1 and Pcl2, as well as by additional cyclins that do not appear to directly regulate G1 events. The G1 cyclins are redundant: cells lacking any two of the cyclins Cln1, Cln2 or Cln3 are viable, but loss of all three cyclins is lethal [2],[3]. Similarly, cells lacking Cln1 and Cln2 or Pcl1 and Pcl2 are viable, but loss of all four cyclins is lethal [4],[5]. The cyclin Cln3 plays a role in triggering transcription of a suite of genes required for initiation of G1 events, including the genes for Cln1, Cln2, and Pcl1, which are often referred to as late G1 cyclins [5]–[9]. Transcription of the late G1 cyclins is generally considered to be the key molecular event that marks entry into the cell cycle [10],[11]. The late G1 cyclins initiate growth of a new daughter bud and are also required for polar growth after bud emergence [4],[12].

Production of late G1 cyclins is tightly regulated. Cyclin mRNA and protein undergo rapid turnover, so mechanisms that act at the level of transcription play an important role [13]–[15]. Transcription of G1-specific genes, including the late G1 cyclin genes, is dependent upon the SBF and MBF transcription factors. SBF and MBF each include a distinct DNA binding subunit, called Swi4 and Mbp1, respectively, and a shared subunit called Swi6. SBF and MBF are kept inactive early in the cell cycle by a repressor called Whi5 [16],[17]. Loss of Whi5 causes transcription of late G1 cyclins to occur before the mother cell has completed growth, leading to premature bud emergence and a reduced cell size. Cdk1/Cln3 triggers transcription of late G1 cyclins by phosphorylating and inactivating Whi5. Transcription of late G1 cyclins can also be triggered by a redundant Cln3-independent pathway that is dependent upon the Bck2 protein [18]–[21]. The late G1 cyclin Cln2 promotes its own transcription via a positive feedback loop, which ensures that initiation of G1 events occurs in a coordinated, switch-like manner [6],[7],[22].

Mechanisms that control G1 cyclin transcription play an important role in control of cell size. A cell size checkpoint links initiation of G1 cyclin transcription to cell size. Thus, transcription of late G1 cyclins is only initiated when the mother cell has reached a critical size, which contributes to cell size homeostasis. An interesting property of cell size control in yeast is that the critical cell size is modulated by external nutrients, such that cells growing in poor nutrients are significantly smaller than cells growing in rich nutrients [23],[24]. It is thought that nutrients modulate cell size by rapidly changing the critical cell size for initiation of G1 cyclin transcription [11].

The mechanisms that link initiation of G1 cyclin transcription to cell size and nutrient availability are unknown. Interestingly, *cln3*Δ *bck2*Δ *whi5*Δ triple mutants, which lack all upstream regulators known to play an important role in the control of G1 cyclin transcription, undergo normal nutrient modulation of cell size [25]. Thus, the signals that control cell size by linking G1 cyclin transcription to nutrient availability must act by a different mechanism. The mechanisms that link G1 cyclin transcription to cell size and nutrient availability are likely to be a key to understanding cell size control.

Here, we report that a specific form of protein phosphatase 2A (PP2A) is required for control of G1 cyclin transcription and nutrient modulation of cell size. PP2A is a trimeric complex that consists of a catalytic “C” subunit, a scaffolding “A” subunit, and a regulatory “B” subunit [26]. Binding of different B-type regulatory subunits is thought to direct PP2A activity toward different substrates. Thus, the key to understanding PP2A is to understand the function and regulation of specific regulatory subunits. In budding yeast, two B subunits called Cdc55 and Rts1 bind to PP2A in a mutually exclusive manner, forming two distinct PP2A complexes: PP2A^Cdc55^ and PP2A^Rts1^ [27],[28].

We discovered a role for Rts1 in controlling G1 cyclin levels while characterizing a genetic interaction between *RTS1* and the septin *CDC12*. The septins are a conserved family of proteins that localize to the site of bud emergence in early G1 and to the bud neck during bud growth and cytokinesis [29]. The septins have been proposed to form a diffusion barrier between the mother and daughter cell, to serve as a signaling scaffold for activation of kinases, or to carry out functions in the secretory pathway. Temperature sensitive alleles of the septins cause cells to undergo a prolonged delay at G2/M while undergoing continuous polarized growth, leading to the formation of highly elongated cells [30]. The G2/M arrest is mediated by Swe1, the budding yeast Wee1 homolog, which phosphorylates and inhibits Cdk1 to delay entry into mitosis [31]. The G2/M delay and the elongated cell phenotype are eliminated by *swe1*Δ. A number of kinases have been identified that regulate septin function and localization, and may in turn be regulated by the septins [31]–[36]. These kinases include Elm1, Gin4, Cla4, and Hsl1. Loss of these kinases can cause a Swe1-dependent G2/M delay and an elongated cell phenotype similar to septin mutants.

Previous work found that *rts1*Δ increased the restrictive temperature of the *cdc12-6* allele [37]. Loss of *RTS1* also caused altered septin ring dynamics; however, it remained unclear whether the observed changes in septin ring dynamics were sufficient to explain the rescue of the *cdc12-6* temperature sensitive phenotype. For example, it was possible that in addition to regulating septin ring dynamics, Rts1 played additional roles in pathways that promote polar growth or Swe1-dependent G2/M delays. We therefore further investigated the role of Rts1 in polar cell growth and cell cycle progression.

## Results

### Loss of the PP2A regulatory subunit Rts1 reduces polar growth caused by loss of septin function

**Figure 1:**
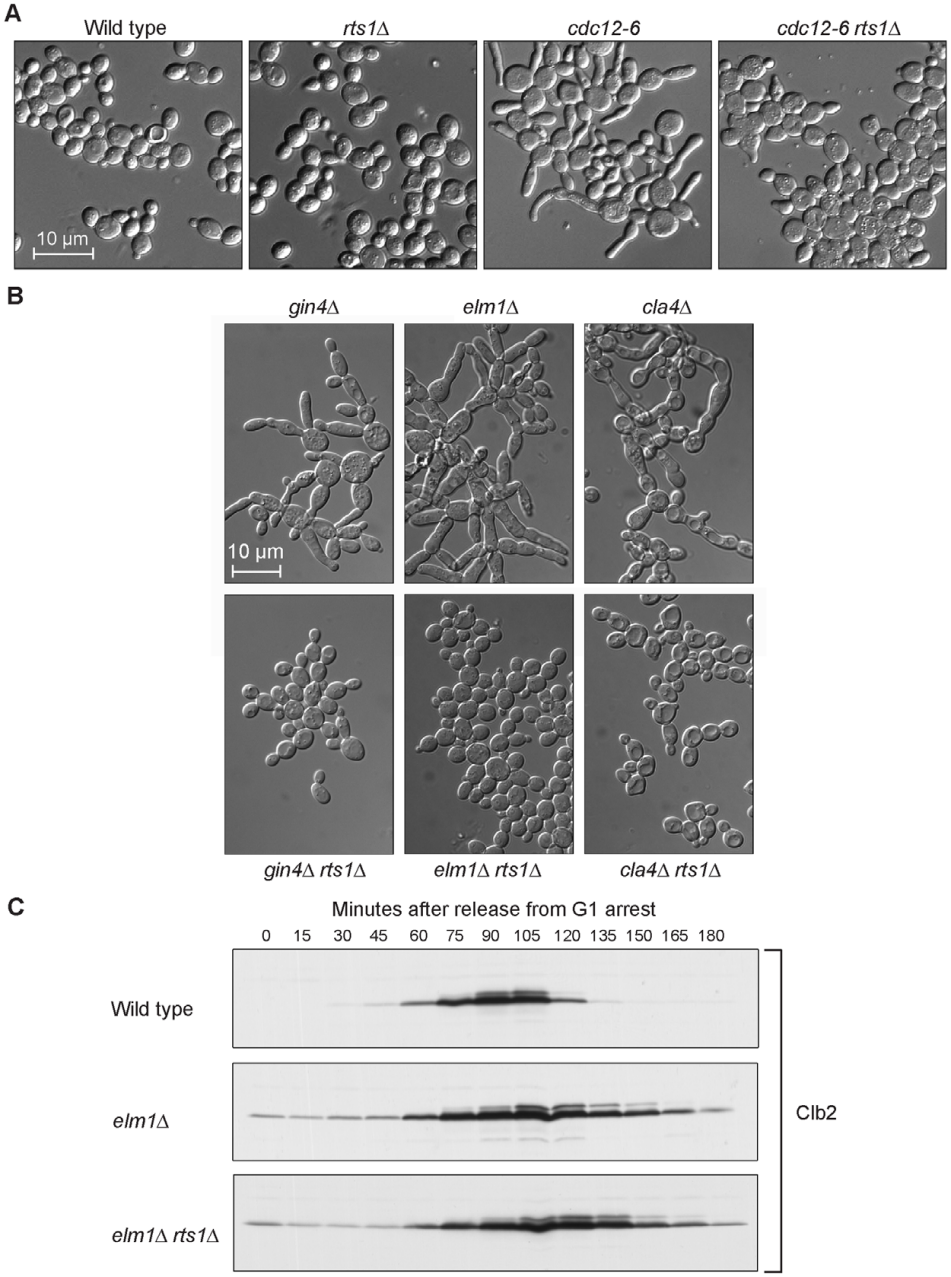
Loss of Rts1 causes reduced polar growth. (A) *rts1*Δ reduces the polar growth caused by inactivation of septins. Cells of the indicated genotypes were grown overnight to log phase at room temperature and then shifted to 30°C for 4 hours. (B) *rts1*Δ reduces the polar growth caused by inactivation of kinases that regulate the septins. Cells of the indicated genotypes were grown to log phase. The *gin4*Δ and *gin4*Δ *rts1*Δ cells were grown at room temperature, while the others were grown at 30°C. (C) *rts1*Δ does not eliminate the G2/M delay caused by *elm1*Δ. Wild type, *elm1*Δ, and *elm1*Δ *rts1*Δ cells were released from an α factor arrest at 30°C. Samples were collected at 10 minute intervals and levels of the mitotic cyclin Clb2 were detected by Western blotting.

Since *rts1*Δ suppressed the temperature sensitivity of *cdc12-6*, we tested whether *rts1*Δ also suppressed the elongated cell phenotype of these cells. We found that *rts1*Δ *cdc12-6* cells showed reduced elongation compared with *cdc12-6* cells when shifted to 30°C (Figure 1A). In addition, *rts1*Δ significantly reduced the elongated cell phenotype caused by loss of *GIN4*, *ELM1*, and *CLA4* (Figure 1B).

We next considered the possibility that *rts1*Δ rescued the elongated cell phenotype of these mutants by eliminating the Swe1-dependent G2/M delay. To test this, levels of the mitotic cyclin Clb2 were assayed by Western blotting during a synchronized cell cycle in wild type, *elm1*Δ, and *elm1*Δ *rts1*Δ cells (Figure 1C). As previously shown, *elm1*Δ cells underwent a prolonged G2/M delay with elevated Clb2 levels when compared to wild type cells [35]. The prolonged G2/M delay was not eliminated by *rts1*Δ. Thus, although *rts1*Δ reduced the elongated cell phenotype caused by loss of *CDC12*, *GIN4*, *CLA4*, and *ELM1*, it did not appear to do so by reducing the Swe1-dependent G2/M delay.

### Rts1 is required for the timely initiation and normal rate of polar cell growth

**Figure 2:**
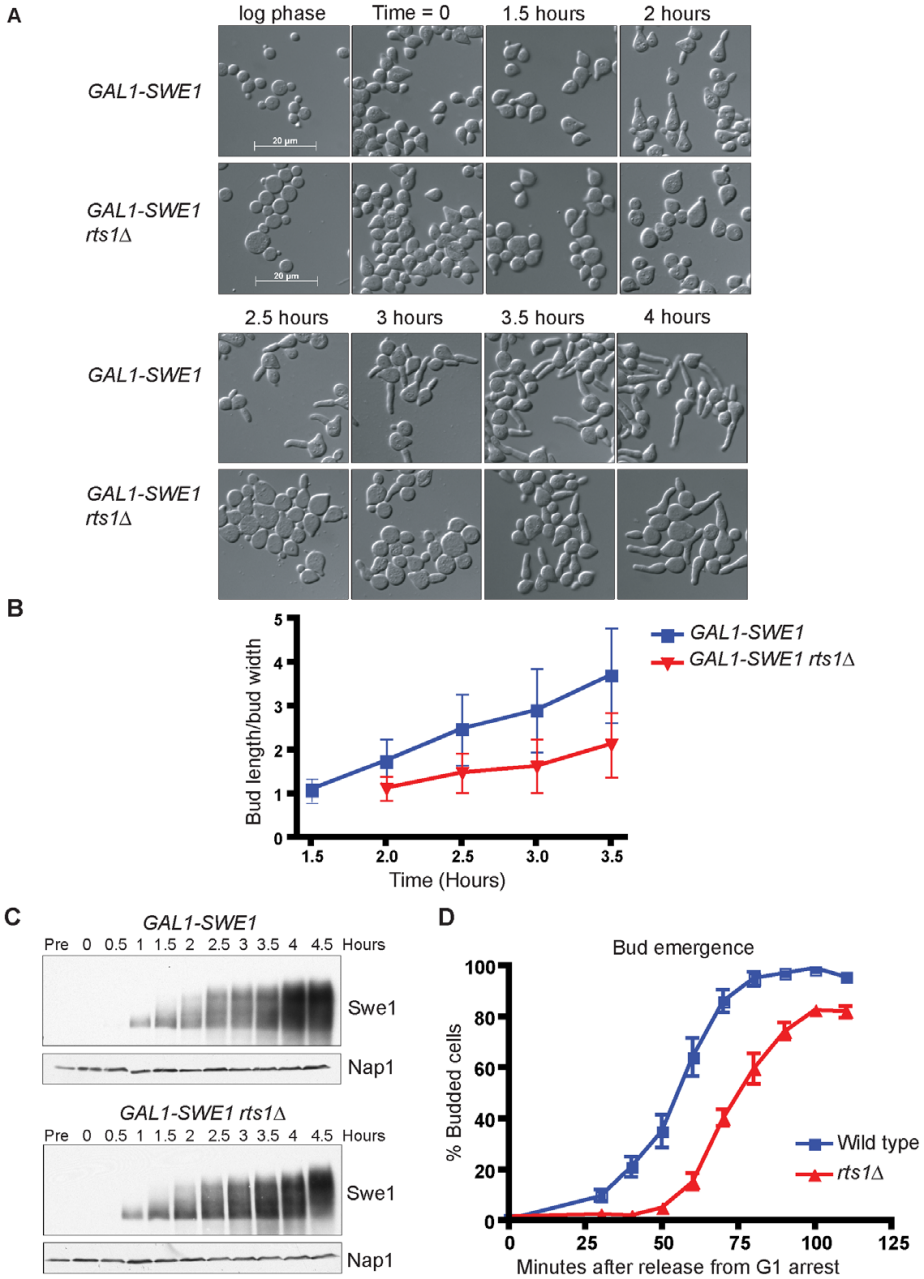
Loss of Rts1 causes a reduced rate of polar bud growth. (A) *rts1*Δ reduces polar growth induced by expression of *SWE1* from the *GAL1* promoter. Cells were grown to log phase in YEP media containing 2% glycerol/ethanol and then arrested in G1 with α factor. The synchronized cells were released into galactose-containing media at 30°C to induce *SWE1* expression, and images of cells were obtained at 30 minute intervals. The time indicates the number of hours after release of α factor-arrested cells into galactose. (B) *rts1*Δ reduces the rate of polar growth in cells that over express *SWE1*. Polar growth was measured and plotted as a function of time for the experiment shown in (A). The extent of polar bud growth was measured as the ratio of the length of the bud to the width of the bud measured at the widest point. The average ratio and standard deviation were plotted for each time point. (C) Western blots showing equal expression of Swe1 protein in wild type and *rts1*Δ cells from the experiment shown in (A). A Nap1 Western blot was included as a loading control. (D) *rts1*Δ causes a delay in bud emergence. Wild-type and *rts1*Δ cells were released from an α factor arrest at 30°C and the percentage of budded cells was determined at 10 minute intervals. Error bars indicate standard deviation for 3 independent experiments.

Since *rts1*Δ did not rescue the G2/M delay in *elm1*Δ cells, we considered the possibility that Rts1 plays a direct role in promoting polar growth. To test this, we utilized cells that over express *SWE1* from the *GAL1* promoter, which arrest at G2/M with high levels of G1 cyclins and undergo constitutive polar growth [12],[38]. Wild type or *rts1*Δ cells carrying *GAL1-SWE1* were released from a G1 arrest in the presence of galactose to induce expression of *SWE1*. We then measured bud lengths at time intervals after induction of *GAL1-SWE1* to determine the rate of polar bud growth. We found that polar bud growth in *GAL1-SWE1 rts1*Δ cells occurred at a slower rate than *GAL1-SWE1* control cells (Figure 2A and 2B). Controls showed that wild type and *rts1*Δ cells expressed similar levels of Swe1 protein (Figure 2C). Similar results were also obtained by measuring rates of bud elongation after induction of *GAL1-SWE1* in log phase populations of cells (not shown).

We next determined whether Rts1 plays a role in initiation of polar bud growth. To do this, we assayed initiation of bud growth in synchronized populations of wild type and *rts1*Δ cells. Cells were released from an early G1 arrest and the timing of bud emergence was determined (Figure 2D). Cells lacking Rts1 showed a delay in bud emergence of 22 minutes ± 0.15 minutes compared with wild type cells. Together, these results demonstrate that Rts1 is required for both the timely initiation and the normal rate of polar bud growth.

### Cells lacking Rts1 are sensitive to the dosage of G1 cyclins

**Figure 3:**
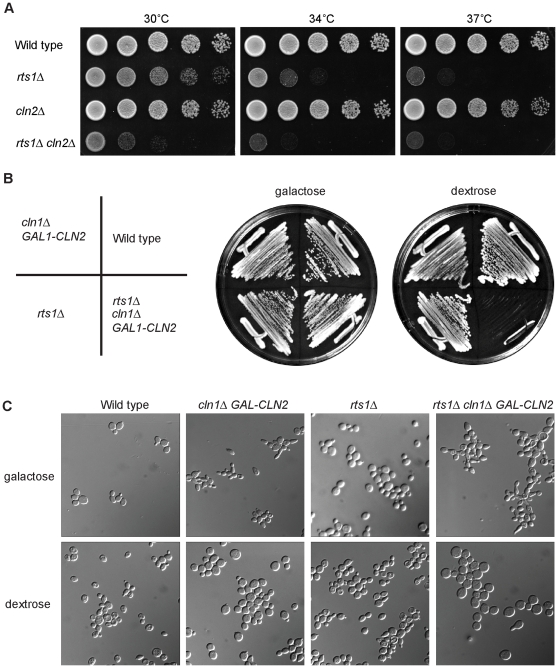
*rts1*Δ cells are sensitive to levels of the G1 cyclins *CLN1* and *CLN2*. (A) *cln2*Δ causes slow growth in *rts1*Δ cells. A series of 10-fold dilutions of cells of the indicated genotypes were grown at 30°C, 34°C, and 37°C. (B) Loss of *CLN1* and *CLN2* is lethal in *rts1*Δ cells. Cells of the indicated genotypes were grown at room temperature for three days at 25°C on YEP media containing galactose or dextrose. (C) Loss of *CLN1* and *CLN2* in *rts1*Δ cells causes increased cell size and an arrest with predominantly unbudded cells. Images of cells of the indicated genotypes were taken 8 hours after expression of *CLN2* was repressed by washing cells out of galactose-containing media into dextrose-containing media at 30°C.

Since G1 cyclins are required for initiation and maintenance of polar bud growth, it seemed likely that Rts1 is required for functions mediated by G1 cyclins. To test this, we determined whether *rts1*Δ showed genetic interactions with the G1 cyclins. We found that *cln2*Δ significantly reduced the rate of proliferation of *rts1*Δ cells (Figure 3A). Moreover, we failed to recover *rts1*Δ *cln1*Δ *cln2*Δ spores when *rts1*Δ was crossed to a *cln1*Δ *cln2*Δ strain, which suggested that *rts1*Δ is synthetically lethal with *cln1*Δ *cln2*Δ. To further test this, we created a *GAL1-CLN2 cln1*Δ *rts1*Δ strain, in which the expression of *CLN2* could be repressed by switching from galactose-containing media to dextrose-containing media. This strain grew well on galactose, but was inviable on dextrose, which confirmed that *rts1*Δ is lethal in *cln1*Δ *cln2*Δ cells (Figure 3B). To characterize the defects caused by loss of Rts1, Cln1, and Cln2, we turned off *CLN2* expression in the *GAL1-CLN2 cln1*Δ *rts1*Δ cells by shifting the cells to media containing dextrose for 8 hours (Figure 3C). The *GAL1-CLN2 cln1*Δ *rts1*Δ cells arrested primarily as large, unbudded cells with a small percentage of budded cells (6.5%). Control cells carrying *rts1*Δ or *cln1*Δ *GAL1-CLN2* had 35% and 15% budded cells respectively. The *GAL1-CLN2 cln1*Δ *rts1*Δ cells also became abnormally large, which is commonly observed in cells that fail to undergo bud emergence (Figure 3C) [39],[40].

We next tested whether *rts1*Δ showed a genetic interaction with *cln3*Δ. When *rts1*Δ cells were crossed to *cln3*Δ cells, a small proportion of the expected progeny was recovered (2/80 spores were recovered rather than the predicted 20/80), while the other progeny segregated according to the expected Mendelian ratios (*rts1*Δ: 21/80, *cln3*Δ: 20/80, wild type: 18/80). The few recovered *rts1*Δ *cln3*Δ cells formed colonies at the same rate as *rts1*Δ cells, but their low rate of recovery from the cross suggested that they could contain suppressor mutations. To analyze the viability of cells lacking *RTS1* and *CLN3* in a context unlikely to select for suppression, we utilized a *GAL1-CLN3 rts1*Δ strain. When switched to dextrose-containing medium we found that *GAL1-CLN3 rts1*Δ cells were viable, although they formed colonies at a slightly slower rate than *rts1*Δ cells (not shown).

**Figure 4:**
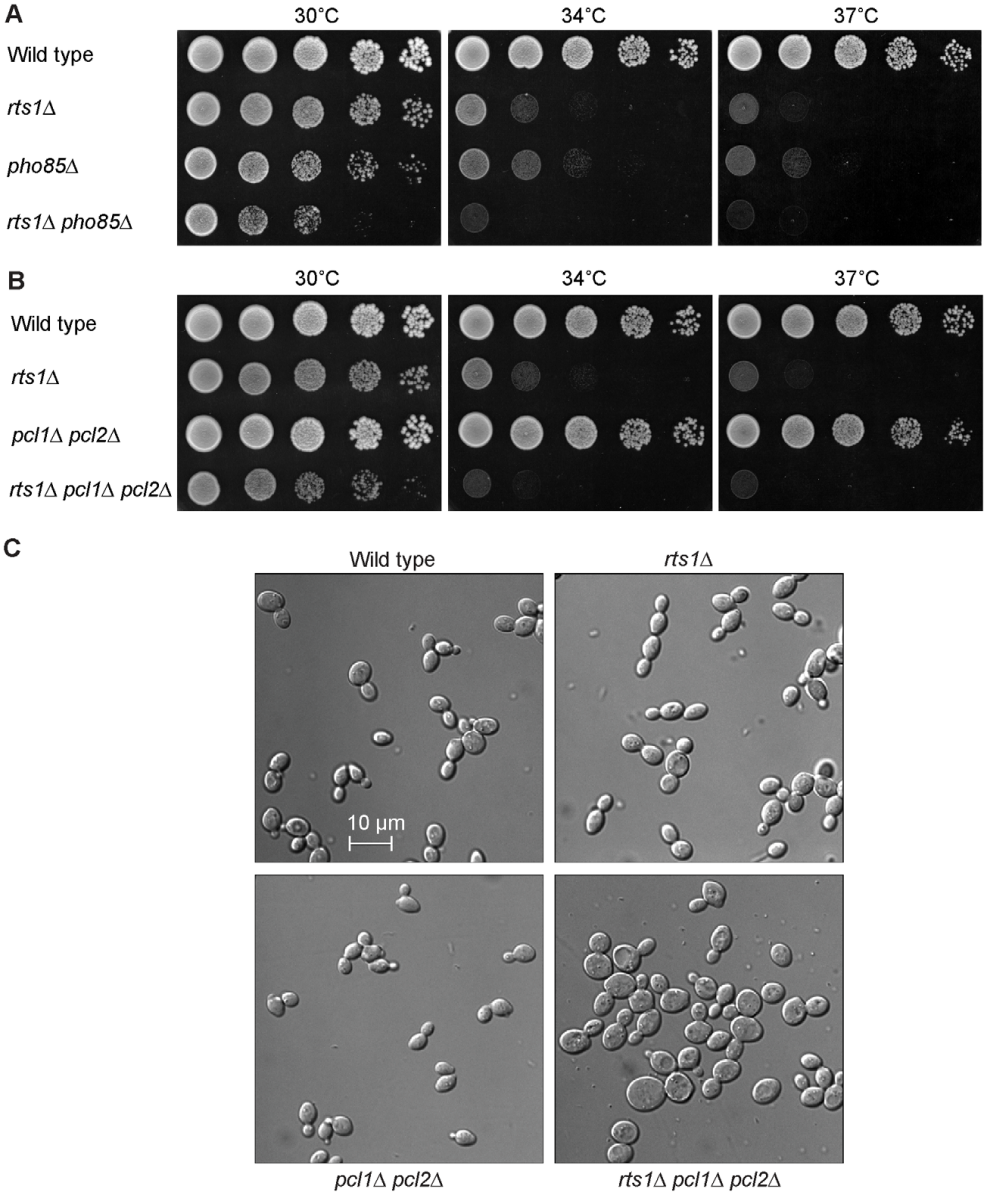
*rts1*Δ causes a reduced rate of proliferation in *pho85*Δ and *pcl1*Δ *pcl2*Δ cells. (A) *pho85*Δ causes slow growth in *rts1*Δ cells. A series of 10-fold dilutions of cells of the indicated genotypes were grown at 30°C, 34°C, and 37°C. (B) *pcl1*Δ *pcl2*Δ causes slow growth in *rts1*Δ cells. A series of 10-fold dilutions of cells of the indicated genotypes were grown at 30°C, 34°C, and 37°C. (C) *rts1*Δ *pcl1*Δ *pcl2*Δ cells are abnormally large. Cells of the indicated genotypes were grown overnight to log phase at 25°C.

It was previously reported that *rts1*Δ is synthetically lethal with the cyclin-dependent kinase Pho85 in the S288C strain background [41]. We were able to isolate a few *rts1*Δ *pho85*Δ spores in the W303 strain background, although they were poorly viable and grew significantly slower than either *rts1*Δ or *pho85*Δ cells (Figure 4A). We were also able to recover *pcl1*Δ *pcl2*Δ *rts1*Δ cells from crosses. These grew more slowly than *rts1*Δ cells but were more robust than *pho85*Δ *rts1*Δ cells (Figure 4B). Thus, the poor viability of *pho85*Δ *rts1*Δ cells is not strictly due to lack of Pho85 activity associated with the Pcl1/2 cyclins, and may indicate that additional Pho85/Pcl complexes are important for normal growth in *rts1*Δ strains. In contrast to cells lacking *CLN1*, *CLN2* and *RTS1*, the *pcl1*Δ *pcl2*Δ *rts1*Δ cells did not show severe defects in bud formation, although they did become larger than the *rts1*Δ or *pcl1*Δ *pcl2*Δ cells. (Figure 4C).

In summary, *rts1*Δ showed genetic interactions with multiple G1 cyclins and cyclin-dependent kinases. Because the late G1 cyclins show extensive redundancy, mutations that cause reduced function of G1 cyclins should show synthetic interactions with mutations that cause a further reduction in cyclin levels. The fact that *rts1*Δ showed lethality when combined with *cln1*Δ *cln2*Δ, and reduced viability when combined with *pcl1*Δ *pcl2*Δ, suggests that Rts1 is required for the normal function of both pairs of cyclins, rather than mediating the functions of specific cyclins. The fact that *rts1*Δ also showed genetic interactions with *cln3*Δ, which is upstream of the late G1 cyclins, indicates that Rts1 does not act solely in a Cln3-dependent pathway that promotes transcription of the late G1 cyclins.

### Rts1 is required for normal accumulation of the G1 cyclins

**Figure 5:**
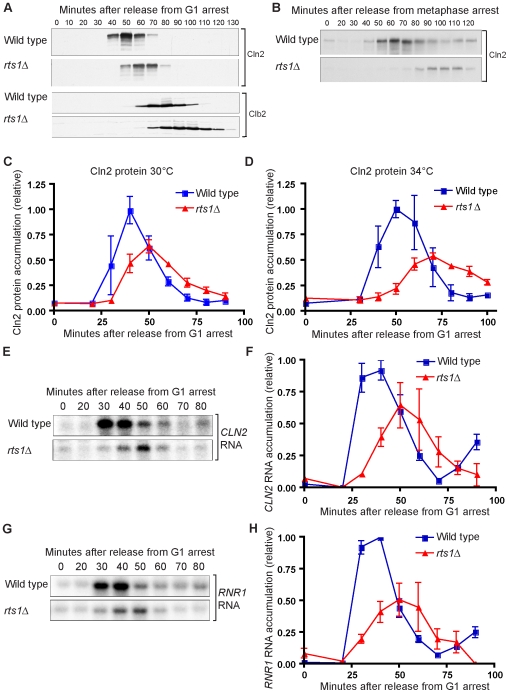
G1 events are delayed in *rts1*Δ cells. (A) Wild-type and *rts1*Δ cells were released from an α factor arrest into pre-warmed media at 30°C. Levels of Cln2-3XHA and Clb2 were monitored by Western blotting. (B) *GAL1-CDC20* and *GAL1-CDC20 rts1*Δ cells were arrested in metaphase by depletion of Cdc20 in raffinose media for 4 hours. Cells were released by addition of galactose. Levels of Cln2-3XHA were monitored by Western blotting. (C) Accumulation of Cln2 protein is delayed and reduced during a synchronized cell cycle in *rts1*Δ cells. Cln2-3XHA protein levels were quantified during a synchronized cell cycle at 30°C. Error bars indicate the standard error of the mean for 3 independent experiments. (D) Accumulation of Cln2 protein is delayed and reduced during a synchronized cell cycle in *rts1*Δ cells. Cln2-3XHA protein levels were quantified during a synchronized cell cycle at 34°C. Error bars indicate the standard error of the mean for 3 independent experiments. (E,F) Accumulation of the *CLN2* mRNA is reduced and delayed during a synchronized cell cycle in *rts1*Δ cells. Wild-type and *rts1*Δ cells were released from an α factor arrest into pre-warmed media at 30°C and samples were collected at 10 minute intervals during the cell cycle. (E) shows levels of *CLN2* mRNA detected by Northern blotting. (F) shows quantification of *CLN2* mRNA normalized to an *ACT1* loading control. Error bars indicate the standard error of the mean for 3 independent experiments. (G,H) Accumulation of the *RNR1* mRNA is reduced and delayed during a synchronized cell cycle in *rts1*Δ cells. Wild type and *rts1*Δ cells were released from an α factor arrest into pre-warmed media at 30°C and samples were collected at 10 minute intervals during the cell cycle. (G) shows levels of *RNR1* mRNA detected by Northern blotting. (H) shows quantification of *RNR1* mRNA normalized to an *ACT1* loading control. Error bars indicate the standard error of the mean for 3 independent experiments.

We next determined whether accumulation of the G1 cyclin Cln2 occurred normally in synchronized *rts1*Δ cells. For these experiments, we utilized a 3XHA-tagged version of *CLN2* expressed from the *CLN2* promoter and quantitative Western blotting to assay Cln2 protein levels. These experiments revealed that the peak of Cln2 accumulation was delayed by 10–15 minutes in *rts1*Δ cells and that Cln2 failed to reach normal levels (Figure 5A and 5C). The effects of *rts1*Δ on Cln2 accumulation were more severe at 34°C and 37°C (Figure 5D). Accumulation of the mitotic cyclin Clb2 was correspondingly delayed and cells appeared to delay in G2/M, as revealed by sustained levels of Clb2 relative to the wild type control (Figure 5A).

Since the cells used in these experiments were synchronized with mating pheromone, it was possible that the delayed accumulation of Cln2 was due to delayed release from mating pheromone arrest. To determine whether *rts1*Δ cells underwent normal release from mating pheromone arrest, we assayed the phosphorylation state of Cdc24, which is the guanine nucleotide exchange factor for Cdc42 [42]. In previous work, it was found that Cdc24 becomes hyperphosphorylated during mating pheromone arrest and undergoes dephosphorylation upon release from the arrest [12],[43]. Cdc24 then undergoes hyperphosphorylation during G1 that is dependent upon the Cla4 kinase and Cdk1 [12],[43],[44]. We found that Cdc24 underwent normal dephosphorylation in *rts1*Δ cells after release from mating pheromone arrest, which suggested that *rts1*Δ does not cause delayed release from mating pheromone arrest (Figure S1A). Cdc24 showed delayed phosphorylation in *rts1*Δ cells, consistent with delayed initiation of G1 events.

To further rule out the possibility that the G1 delay was due to mating pheromone-induced arrest, we used an alternative method for cell synchronization. Cells can be arrested in mitosis by depletion of Cdc20, which is required for proteolytic destruction of the mitotic cyclins [45]–[47]. *CDC20* was placed under the control of the *GAL1* promoter in wild type and *rts1*Δ cells. Synchronization in metaphase was achieved by shifting cells to media lacking galactose for 4 hours, followed by releasing cells into galactose-containing media to initiate synchronous exit from mitosis. Cells lacking Rts1 showed a 30–40 minute delay in Cln2 accumulation and reduced Cln2 levels under these conditions (Figure 5B).

We also tested whether the effects of *rts1*Δ on Cln2 accumulation were dependent upon the strain background. Several commonly used laboratory yeast strains contain different alleles of the *SSD1* gene, which can cause significant differences in phenotypes [48]. However, we found that *rts1*Δ caused similar defects in Cln2 accumulation in both the W303 (*ssd1-d2*) strain background and the S288C (*SSD1*-*v1*) strain background (Figure 5 and Figure S1B, respectively).

Our finding that Cln2 accumulation was delayed and reduced in *rts1*Δ cells suggested an explanation for the reduced polar growth caused by *rts1*Δ in mutants that undergo excessive polar growth (Figure 1A and 1B and Figure 2A). We hypothesized that *rts1*Δ leads to reduced and delayed Cln2 accumulation in these cells, thereby causing reduced polar growth. We tested this directly by assaying Cln2 accumulation in synchronized *gin4*Δ and *rts1*Δ *gin4*Δ cells. As expected, rts1Δ caused reduced and delayed accumulation of Cln2, and a corresponding delay in Clb2 accumulation (Figure S2).

We next tested whether *rts1*Δ affected levels of *CLN2* mRNA or mRNAs encoding additional G1 cyclins. Northern blotting revealed that accumulation of *CLN2*, *CLN1* and *PCL1* mRNA was reduced and delayed in *rts1*Δ cells (Figure 5E and 5F and Figure S1C). Transcription of the late G1 cyclins is controlled by the SBF transcription factor. To test whether *rts1*Δ caused delayed transcription of MBF targets as well, we assayed *RNR1* mRNA expression. *RNR1* mRNA accumulation was reduced and delayed to a similar extent as *CLN2* mRNA in *rts1*Δ cells (Figure 5G and 5H). Together these results show *rts1*Δ causes reduced and delayed accumulation of both SBF and MBF-regulated transcripts.

### Expression of *CLN2* from a heterologous promoter rescues the delayed bud emergence of *rts1*Δ cells

**Figure 6:**
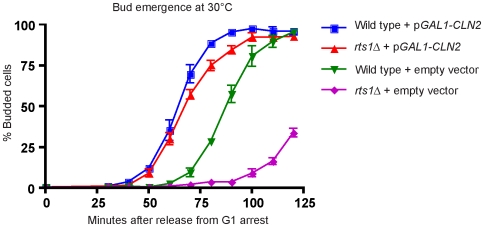
Expression of *CLN2* from a heterologous promoter rescues the delayed bud emergence of *rts1*Δ cells. Wild-type and *rts1*Δ cells carrying *GAL1-CLN2* or an empty vector were released from an α factor arrest at 30°C and the percentage of budded cells was determined at 10 minute intervals. Over 200 cells were counted for each time point. Error bars indicate the standard error of the mean for 3 independent experiments.

Since *rts1*Δ caused reduced and delayed transcription of G1 cyclins, the delayed bud emergence observed in *rts1*Δ cells could be due solely to a role for Rts1 in promoting G1 cyclin transcription. Alternatively, Rts1 could play diverse roles in regulating events required for bud emergence. To distinguish these possibilities, we tested whether expression of *CLN2* from the *GAL1* promoter could rescue the delayed bud emergence of *rts1*Δ cells. Wild type and *rts1*Δ cells carrying *GAL1-CLN2* or an empty vector were released from a G1 arrest under conditions that induce expression of *CLN2*, and the timing of bud emergence was assayed. We found that expression of *CLN2* from the *GAL1* promoter dramatically advanced the timing of bud emergence in *rts1*Δ cells, providing nearly complete rescue of the delay in bud emergence (Figure 6). This observation, combined with our previous observations, established that Rts1 functions in mechanisms directly involved in controlling transcription of the G1 cyclins. Expression of *GAL1-CLN2* did not rescue the temperature sensitivity of *rts1*Δ cells, which indicated that the temperature sensitivity of *rts1*Δ cells must be due, at least in part, to additional functions of Rts1 (not shown).

### Rts1 does not regulate the turnover of Cln2 protein

Recent work found that Cln2 acts in a positive feedback loop to stimulate its own transcription [22]. Thus, the delay in accumulation of *CLN2* mRNA could be due to a failure in mechanisms required for normal accumulation of the Cln2 protein, which would disrupt the feedback loop. Overexpression of *CLN2* from the *GAL1* promoter might be expected to rescue this kind of defect. Therefore, we tested whether Rts1 functions in several mechanisms known to regulate accumulation of *CLN2* protein.

**Figure 7:**
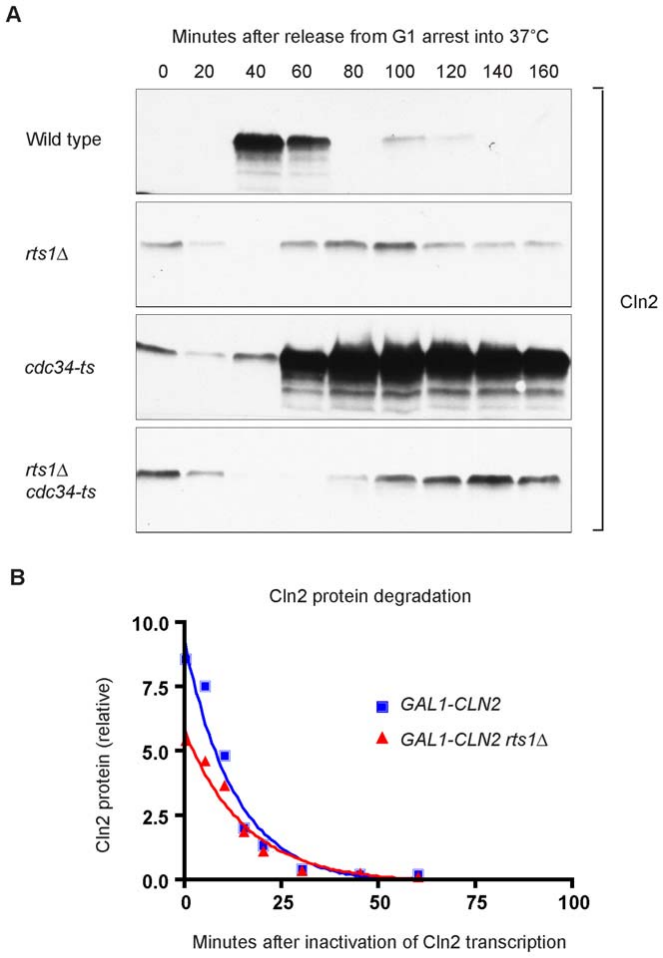
The half-life of Cln2 is not reduced in *rts1*Δ cells. (A) Inactivation of the SCF E3 ubiquitin ligase does not cause increased Cln2 protein levels in *rts1*Δ cells. Cells of the indicated genotypes were released from an α factor arrest into pre-warmed media at 37°C. Levels of Cln2-3XHA protein were monitored by Western blotting. (B) *rts1*Δ does not decrease the half-life of the Cln2 protein. *GAL1-3XHA-CLN2* and *GAL1-3XHA-CLN2 rts1*Δ cells were grown overnight at room temperature in YEP media containing 2% glycerol/ethanol. A burst of *CLN2* transcription was triggered by washing the cells into YEP +2% galactose for 1 hour. At time = 0, *CLN2* transcription was turned off by washing cells into YEPD media. Samples were collected at t = 0, 5, 10, 15, 20, 30, 45, 60, and 120 minutes, and quantitative Western blotting was used to determine Cln2 levels. The half-life of Cln2 protein was calculated using a standard curve fitted for one phase exponential decay. The half-life of Cln2 was 9.6±2.0 minutes in wild type cells and 10.8±2.4 minutes in *rts1*Δ cells (n = 3).

Cln2 is a highly unstable protein and proteolysis plays an important role in regulation of Cln2 protein levels. Proteolysis of Cln2 is controlled by the SCF ubiquitin ligase complex, which recognizes phosphorylated sites at the C-terminus of Cln2 and targets Cln2 for destruction [13],[49]. Cdc34 is the E2 ubiquitin conjugating enzyme component of the SCF complex. We hypothesized that the reduced Cln2 protein levels observed in *rts1*Δ cells could be caused by increased SCF-dependent proteolysis of Cln2 protein. Reduced protein levels, in turn, would disrupt the positive feedback loop that promotes *CLN2* transcription, thereby causing reduced and delayed transcription of *CLN2* mRNA. To test this possibility, we created an *rts1*Δ strain that also contained a temperature sensitive allele of *CDC34* (*cdc34-2*). Cells were released from a synchronized G1 arrest into 37°C media, and Cln2 protein expression was followed by Western blotting (Figure 7A). As previously reported, inactivation of Cdc34 in the control cells caused stabilization of Cln2 and a dramatic increase in Cln2 protein levels [13]. In the *cdc34-2 rts1*Δ cells, Cln2 protein levels were still reduced and did not accumulate to the high levels observed in *cdc34-2* cells. This showed that the failure of *rts1*Δ cells to accumulate normal levels of Cln2 is due to a failure to produce Cln2, rather than to increased SCF-dependent destruction of Cln2.

We also considered the possibility that Rts1 regulates Cln2 stability via SCF-independent mechanisms. The Cln2 protein has a short half-life of 8–10 minutes [13],[49]. To determine whether *rts1*Δ decreased the half-life of the Cln2 protein, we expressed a burst of *CLN2* from the *GAL1* promoter and then measured the rate of destruction of Cln2 protein after shutting off the promoter. In wild type control cells, the half-life of Cln2 was 9.6±2.0 minutes, similar to previous reports. In *rts1*Δ cells, the half-life of Cln2 was 10.8±2.4 minutes, which showed that there is not a significant decrease in the stability of Cln2 protein (Figure 7B).

### Rts1 does not act in a Whi5-dependent pathway

**Figure 8:**
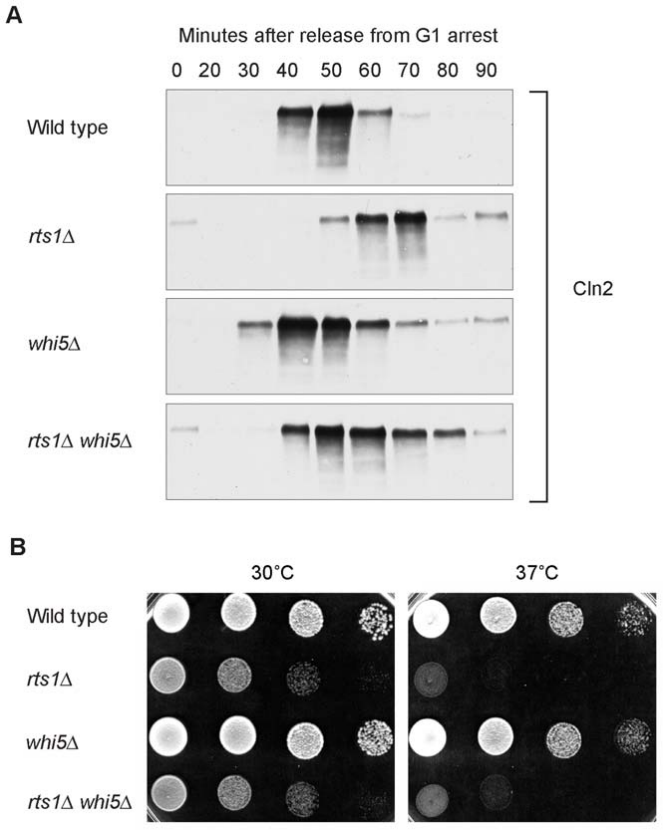
Rts1 acts by a Whi5-independent mechanism. (A) *rts1*Δ causes a G1 delay in *whi5*Δ cells. Cells of the indicated genotypes were released from an α factor arrest into pre-warmed media at 34°C and samples were collected at 10 minute intervals. Levels of Cln2-3XHA were assayed by Western blotting. (B) *whi5*Δ does not rescue the temperature sensitivity of *rts1*Δ. A series of 10-fold dilutions of cells of the indicated genotypes were grown at 30°C and 37°C.

To further define the function of Rts1, we tested whether it acts in pathways known to control G1 cyclin transcription. The Whi5 transcriptional repressor delays G1 progression by inhibiting transcription of G1 cyclins. Whi5 acts by binding and inhibiting the SBF and MBF transcription factors, which are required for transcription of the G1 cyclins [16],[17]. The Cdk1/Cln3 complex relieves this inhibition by phosphorylating Whi5, which triggers export of Whi5 from the nucleus. Thus, it was possible that Rts1 played a role in the inactivation of Whi5. If this were true, *whi5*Δ should rescue the delayed expression of Cln2 observed in *rts1*Δ cells. However, we found that bud emergence and accumulation of Cln2 protein were still delayed in *rts1*Δ *whi5*Δ cells compared to *whi5*Δ cells (Figure 8A, and data not shown). In addition, *whi5*Δ did not rescue the temperature sensitivity of *rts1*Δ cells (Figure 8B). Loss of Whi5 advanced the production of Cln2 protein in *rts1*Δ cells, although not to the same extent observed in *whi5*Δ cells, which indicated that Whi5-dependent regulation of transcription occurs normally in *rts1*Δ cells. Thus, the delayed Cln2 expression in *rts1*Δ cells is not due to a failure to inactivate Whi5.

### Rts1 does not function solely in a Bck2-dependent pathway

Bck2 acts in a redundant pathway that works in parallel to Cln3 to promote transcription of G1 cyclins [18]–[20]. To test whether Rts1 acts in this Bck2-dependent pathway, we crossed *rts1*Δ to *bck2*Δ to create *rts1*Δ *bck2*Δ cells. If Rts1 functioned solely in the Bck2-dependent pathway, we expected to see no additive phenotypic effects in the double mutant.

**Figure 9:**
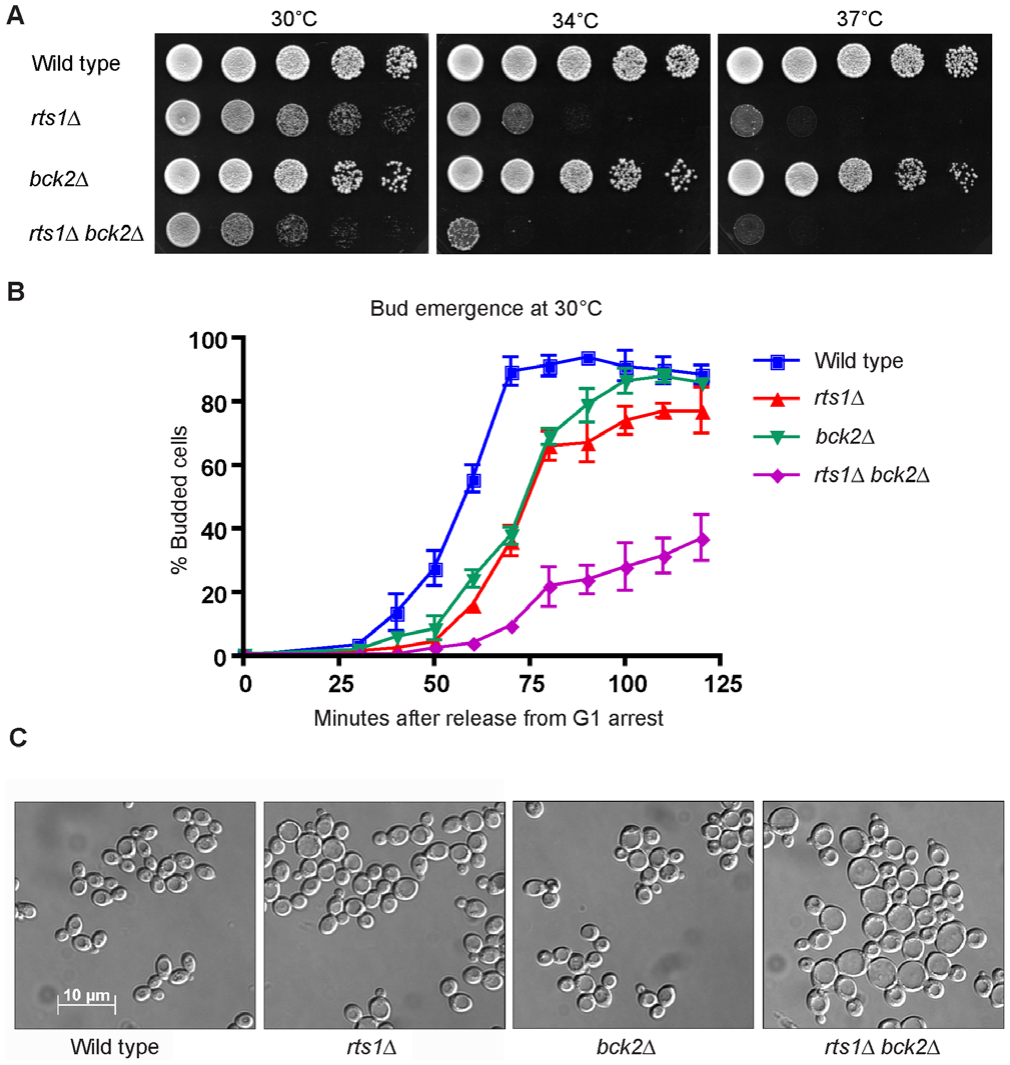
*rts1*Δ shows additive defects with *bck2*Δ. (A) *rts1*Δ causes slow growth in *bck2*Δ cells. A series of 10-fold dilutions of cells of the indicated genotypes were grown at 30°C, 34°C, and 37°C. (B) *rts1*Δ causes a prolonged delay in bud emergence in *bck2*Δ cells. Cells were released from an α factor arrest at 30°C and the percentage of cells with buds was determined at 10 minute intervals. A minimum of 200 cells was counted for each time point. Error bars indicate the standard error of the mean for 3 independent experiments. (C) *rts1*Δ causes increased cell size in *bck2*Δ cells. Images of cells from rapidly growing cultures of cells of the indicated genotypes. Cells were grown at 25°C.

All of the expected *rts1*Δ *bck2*Δ progeny were recovered from the cross. We found that *bck2*Δ increased the temperature sensitivity of *rts1*Δ (Figure 9A). To test whether deletion of Bck2 altered the timing of G1 events in *rts1*Δ cells, we compared the timing of bud emergence and Cln2 accumulation in wild type, *rts1*Δ, *bck2*Δ, and *rts1*Δ *bck2*Δ cells. Cells lacking *BCK2* delayed bud emergence to a similar extent as *rts1*Δ cells. Bud emergence was severely delayed in *rts1*Δ *bck2*Δ cells when compared to either single mutant, and a subset of cells failed to bud by 2 hours after release from G1 arrest (Figure 9B). Cln2 accumulation peaked later in *rts1*Δ *bck2*Δ cells than in *rts1*Δ or *bck2*Δ cells, and accumulated to lower levels (not shown). These observations are consistent with previous reports that *CLN2* mRNA is reduced in *bck2*Δ cells [18],[19]. When tested at 34°C, we saw results that were similar, although more severe (not shown). We examined the phenotype of *rts1*Δ *bck2*Δ cells in log phase cultures that were grown at room temperature, and found that *rts1*Δ *bck2*Δ cells appeared much larger than either *rts1*Δ or *bck2*Δ cells (Figure 9C). The strong additive effects of *rts1*Δ and *bck2*Δ rule out the possibility that Rts1 functions solely in the Bck2-dependent pathway that controls G1 cyclin transcription, although it remains possible that Rts1 contributes to both Bck2-dependent and independent pathways.

### Rts1 may regulate the Swi6 component of SBF and MBF

**Figure 10:**
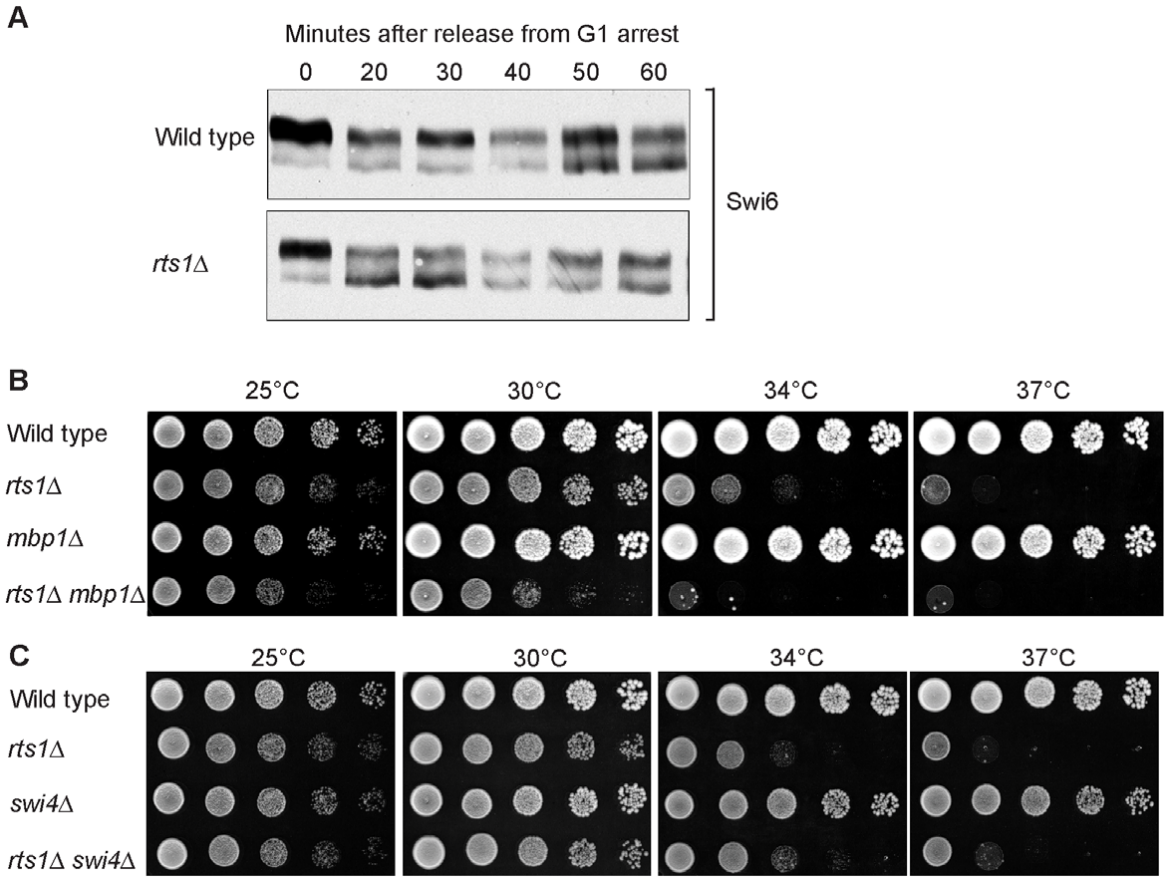
Rts1 is required for normal phosphorylation of Swi6. (A) Swi6 is dephosphorylated in *rts1*Δ cells at the time of initiation of G1 cyclin transcription. Wild type and *rts1*Δ cells were released from an α factor arrest into pre-warmed media at 30°C. Swi6-3XHA modification was monitored by Western blotting. (B,C) *mbp1*Δ but not *swi4*Δ causes a reduced growth rate in *rts1*Δ cells. A series of 10-fold dilutions of cells of the indicated genotypes were grown at 25°C, 30°C, 34°C, and 37°C.

We next determined whether we could detect a role for Rts1 in regulating SBF or MBF. The components of SBF and MBF are Swi6, Swi4 and Mbp1. The Stb1 protein also associates with SBF and MBF and regulates their activity [50]. We therefore generated 3XHA-tagged versions of these proteins and determined whether they showed Rts1-dependent changes in modification state or protein levels. Stb1-3XHA and Swi6-3XHA showed multiple forms on Western blots due to phosphorylation, as previously reported [50]–[52]. Loss of Rts1 caused no detectable changes in the levels of Stb1 modification during a synchronized cell cycle (not shown). In contrast, Swi6-3XHA showed reduced phosphorylation in *rts1*Δ cells at 20 to 30 minutes after release from a mating pheromone arrest (Figure 10A). Notably, the defect in Swi6 phosphorylation occurred at the time that cells would normally be initiating G1 cyclin transcription. We detected no changes in the protein levels of Swi4 or Mbp1 in *rts1*Δ cells during the cell cycle. Swi4 and Mbp1 both migrated as a single band, so electrophoretic mobility shifts could not be used to assay their modification states.

We also tested for genetic interactions between *rts1*Δ and *swi6*Δ, *swi4*Δ or *mbp1*Δ. Previous studies found that *swi6*Δ is recovered poorly from genetic crosses (27% of expected *swi6*Δ progeny are recovered) [53]. We obtained similar results, and were unable to obtain *swi6*Δ *rts1*Δ progeny, suggesting that *swi6*Δ is synthetically lethal with *rts1*Δ. We found that *mbp1*Δ *rts1*Δ cells grew more poorly than either single mutant (Figure 10B). We detected no genetic interaction with *swi4*Δ (Figure 10C). The synthetic lethal interaction with *swi6*Δ must be treated with caution because *swi6*Δ shows synthetic lethality with a surprisingly broad range of genes, including genes that do not appear to have G1-specific functions (BioGRID database). Thus, the synthetic lethality may be due to functions of Rts1 that are not related to G1 functions.

### Rts1 is required for nutrient modulation of cell size

**Figure 11:**
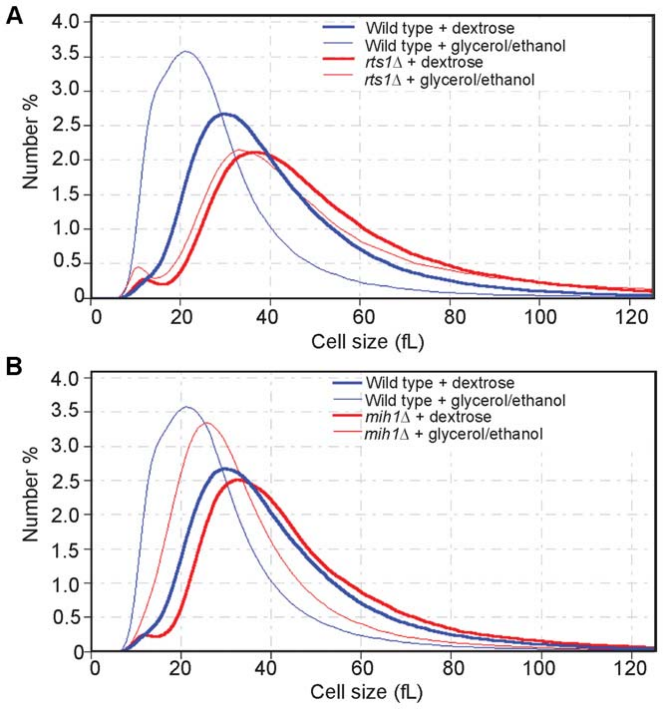
Rts1 is required for nutrient modulation of cell size. Wild-type, *rts1*Δ (A), and *mih1*Δ (B) cells were grown to log phase in media containing either 2% dextrose or 2% glycerol/ethanol as a carbon source. Cell size was measured with a Coulter counter and plotted as a distribution of number % of cells in each size bin. Each plot is the average of 4 independent experiments in which 3 independent samples were analyzed.

Our analysis of the role of Rts1 in control of G1 cyclins suggested that Rts1 does not function solely in the known critical pathways for control of G1 cyclin transcription that work through Cln3, Whi5, or Bck2. This was an intriguing discovery, because previous work found that the mechanisms responsible for nutrient modulation of cell size do not control G1 cyclin transcription via Cln3, Whi5 or Bck2 [11],[25]. We therefore hypothesized that Rts1 controls G1 cyclin transcription in a distinct pathway that mediates nutrient modulation of cell size. To test this, we determined whether Rts1 is required for nutrient modulation of cell size. We grew wild type and *rts1*Δ cells in rich or poor carbon sources and measured cell size with a Coulter counter. We found that Rts1 is required for nutrient modulation of size (Figure 11A). The slight shift in size observed for *rts1*Δ cells shifted to poor carbon sources is similar to the slight shift observed for *sch9*Δ and *sfp1*Δ, which have also been found to be required for nutrient modulation of cell size [25]. Since *rts1*Δ cells are abnormally large, we were concerned that they may already be above the critical size for initiation of G1 cyclin transcription, and therefore not subject to G1 size control. To test this, we used *mih1*Δ cells, which are abnormally large because they undergo extra growth during G2/M [54],[55]. The *mih1*Δ cells showed normal nutrient modulation of cell size control (Figure 11B). Furthermore, others have found that *cln3*Δ cells, which are also abnormally large, undergo normal nutrient modulation of cell size [25].

## Discussion

### Rts1 is required for normal control of G1 cyclin transcription

Loss of Rts1 caused reduced and delayed expression of multiple G1 cyclins, and a corresponding delay in bud emergence. Overexpression of *CLN2* from a heterologous promoter largely rescued the delayed bud emergence in *rts1*Δ cells. We found no evidence that Rts1 regulates Cln2 protein stability. Together, these observations demonstrate that Rts1 functions in a pathway that regulates G1 cyclins at the level of transcription.

The results of genetic analysis further supported the conclusion that Rts1 works in a pathway that controls G1 cyclin levels. The genetic interactions that we observed for *rts1*Δ are summarized in Table 1. In general, *rts1*Δ caused slow growth or lethality when combined with deletions of G1 cyclin genes or *pho85*Δ. Due to the redundancy of the G1 cyclins, a general reduction in levels of G1 cyclin expression caused by *rts1*Δ would be expected to cause additive effects when combined with gene deletions that further reduce levels of G1 cyclins.

**Table 1:** Summary of genetic interactions.

Genotype	Viability		
	30°C	34°C	37°C
Wild type	+++	+++	+++
*rts1*Δ	++	+/−	−
*cln2*Δ	+++	+++	+++
*cln2*Δ *rts1*Δ	+/−	−	−
*cln1*Δ *cln2*Δ *rts1*Δ	−	−	−
*pho85*Δ	++	+	+/−
*pho85*Δ *rts1*Δ	+	−	−
*pcl1*Δ *pcl2*Δ	+++	+++	++
*pcl1*Δ *pcl2*Δ *rts1*Δ	++	−	−
*whi5*Δ	+++	n/a	+++
*whi5*Δ *rts1*Δ	++	n/a	−
*bck2*Δ	+++	+++	++
*bck2*Δ *rts1*Δ	+	−	−
*swi4*Δ	+++	+++	+++
*swi4*Δ *rts1*Δ	++	+/−	−
*swi6*Δ *rts1*Δ	−	−	−
*mbp1*Δ	+++	+++	+++
*mbp1*Δ *rts1*Δ	+	−	−

n/a = Not tested.

The finding that Rts1 is required for normal levels of Cln2 protein provides an explanation for why *rts1*Δ caused reduced polar growth in mutants that fail to properly inactivate Swe1. Failure to inactivate Swe1 causes cells to arrest at G2/M with high levels of Cln2 protein [12]. Since Cln2 promotes polar growth, a reduction in Cln2 levels would be expected to cause reduced polar growth. Accordingly, we found that *rts1*Δ caused reduced and delayed accumulation of Cln2 in *gin4*Δ cells, which fail to inactivate Swe1.

Previous work found that G1 cyclin transcription is regulated by another PP2A-like phosphatase called Sit4. Loss of Sit4 caused decreased transcription of late G1 cyclins and defects in bud emergence, similar to *rts1*Δ [56]. However, there are a number of significant differences in the G1 phenotypes caused by *sit4*Δ and *rts1*Δ. First, in contrast to *rts1*Δ, defects in bud emergence caused by *sit4*Δ are not rescued by expression of *CLN2* from a heterologous promoter, which demonstrates that Sit4 carries out functions required for bud emergence beyond controlling G1 cyclin transcription [56]. Second, the phenotype of *sit4*Δ cells is strongly enhanced by the *ssd1-d2* allele, whereas the phenotype of *rts1*Δ is not affected by the status of *SSD1* [56]. Finally, *sit4*Δ *cln3*Δ cells are barely viable, whereas loss of *RTS1* caused relatively mild effects in *cln3*Δ cells [56]. These phenotypic differences suggest that Rts1 and Sit4 may function in independent pathways that regulate late G1 cyclin levels in response to different signals, but do not rule out the possibility that they have overlapping functions.

### Rts1 does not function solely in one of the pathways known to play a critical role in transcription of G1 cyclins

We used epistasis analysis to determine whether Rts1 regulates G1 cyclin transcription via known mechanisms. This analysis demonstrated that Rts1 does not function solely in a Bck2-dependent pathway, and ruled out a role for Rts1 in a pathway known to regulate G1 cyclin transcription via Cln3-dependent inactivation of the Whi5 transcriptional repressor. Cln3 may also promote G1 cyclin transcription in a Whi5-independent manner. Overexpression of *CLN3-1* makes *whi5*Δ cells smaller, which suggests that Cln3 can drive G1 cyclin transcription by an alternative mechanism [16]. In genetic crosses, we found that most *cln3*Δ *rts1*Δ spores failed to germinate, and cells lacking both Rts1 and Cln3 showed slow growth when compared to either single deletion. Thus, Rts1 does not appear to function solely in a Cln3-dependent pathway that promotes G1 cyclin transcription. Together, these observations suggest that Rts1 does not function solely in one of the known pathways that play an important role in promoting G1 cyclin transcription. Our results do not rule out the possibility that Rts1 contributes to multiple pathways.

### Rts1 may regulate SBF and MBF via Swi6

We found that *rts1*Δ caused reduced and delayed expression of both SBF and MBF-dependent transcripts, which demonstrated that Rts1 acts in a pathway upstream of both transcription factors. We further found that *rts1*Δ caused a reduction in Swi6 phosphorylation at the time that cells initiate G1 cyclin transcription. Since Swi6 is the one shared component of SBF and MBF, this suggests that Rts1 regulates both transcription factors via Swi6. In support of this, the pattern of genetic interactions observed for *rts1*Δ is similar to the pattern previously reported for *swi6*Δ. Both *swi6*Δ and *rts1*Δ cause slow growth in *cln2*Δ cells, lethality in *cln1*Δ *cln2*Δ cells, and slow growth or lethality in *bck2*Δ cells [57],[58]. In addition, neither *rts1*Δ nor *swi6*Δ caused synthetic lethality in combination with *cln3*Δ cells [58]. In contrast, loss of *SWI4* is lethal in combination with *cln3*Δ [58]. The fact that Swi6 undergoes reduced phosphorylation in *rts1*Δ cells, rather than hyperphosphorylation, indicates that it is unlikely to be a direct target of PP2A^Rts1^.

Phosphorylation of Swi6 that can be detected by an electrophoretic mobility shift is dependent upon the MAP kinase Slt2, and activation of Slt2 coincides with initiation of polar growth [51],[59]. The Slt2 pathway is activated by Pkc1, and overexpression of Pkc1 suppresses *swi4* mutants, which demonstrates a role in controlling G1 cyclin transcription [60]. In previous work, *slt2*Δ was not found to cause reduction in the levels of *CLN1* or *CLN2* transcripts, but did cause a reduction in levels of *PCL1* and *PCL2* transcripts in cells grown at 37°C. These studies did not utilize synchronized cells and may therefore have missed effects of *slt2*Δ on levels of *CLN1* and *CLN2* transcripts. In addition, *rts1*Δ could lead to misregulation of Slt2 that causes effects on *CLN1* and *CLN2* transcripts that are more significant than the effects caused by *slt2*Δ. Further work will be necessary to test for possible roles of Rts1 in Slt2-dependent regulation of G1 cyclin transcription.

### Rts1 is required for nutrient modulation of cell size

Previous work found that nutrient modulation of cell size does not require Cln3, Whi5, or Bck2, which suggests that it works via a distinct mechanism. The discovery that Rts1 does not function solely in one of the pathways known to play a critical role in controlling G1 cyclin transcription therefore prompted us to test whether Rts1 is required for nutrient modulation of cell size. We found that Rts1 is required for nutrient modulated control of cell size, which identifies Rts1 as a new component of the pathways that integrate nutrient availability, cell size, and entry into the cell cycle.

Little is known about the pathways responsible for nutrient modulation of cell size. Two conserved pathways play broad roles in controlling nutrient sensing, nutrient utilization, cell growth and cell cycle entry [61]. In one pathway, the TOR kinases respond to the availability of nitrogen and trigger activation of pathways that control cell growth and nitrogen utilization. A second pathway responds to carbon source availability and regulates growth via activation of the Ras GTPase and protein kinase A (PKA) [61]. The Ras/PKA pathway is required for control of cell size: increased activity of the pathway leads to increased cell size, while decreased activity leads to reduced cell size [62]–[64]. However, the mechanisms by which Ras/PKA control cell size are unknown.

A key target of both pathways is ribosome biogenesis. Two key regulators of ribosome biogenesis that are thought to regulate cell growth are Sch9 and Sfp1. Sch9 is a member of the AGC kinase family and is closely related to vertebrate Akt kinase, while Sfp1 is related to transcription factors [65]. Sch9 and Sfp1 carry out overlapping functions in controlling transcription of genes required for ribosome biogenesis [25],[66],[67]. Loss of either protein causes reduced ribosome biogenesis, while loss of both is lethal. The TOR pathway appears to work through Sch9, but the mechanisms by which the Ras/PKA pathway controls ribosome biogenesis are unclear [65],[68].

It is thought that nutrient modulation of cell size is achieved by changing the critical cell size required for initiation of G1 cyclin transcription [11]. Thus far, only three proteins have been found to be required for nutrient modulation of cell size: Sch9, Sfp1 and PKA [25],[64]. Notably, all three converge on control of ribosome biogenesis. Moreover, mutants that cause reduced rates of ribosome biogenesis cause cells to enter the cell cycle at a reduced cell size, leading to an overall reduction in cell size [67]. Together, these observations suggest a model in which the rate of ribosome biogenesis sets the critical cell size [11],[67]. According to this model, cells growing in rich nutrients have a high rate of ribosome biogenesis, which sends a signal that inhibits G1 cyclin transcription to allow the cell to reach a larger critical size.

The mechanisms that link initiation of G1 cyclin transcription to ribosome biogenesis and nutrient availability are unknown. The identification of Rts1 as a new upstream regulator of G1 cyclin transcription that is also required for nutrient modulation of cell size is therefore a significant step towards understanding these pathways. Rts1 could function between ribosome biogenesis and G1 cyclin transcription. Alternatively, Rts1 could promote ribosome biogenesis, in which case the effects of *rts1*Δ could be due to decreased rates of ribosome biogenesis. Inactivation of factors that promote ribosome biogenesis cause reduced cell size, whereas *rts1*Δ causes increased cell size. However, we do not yet know what causes *rts1*Δ cells to have an increased cell size, and it could be due to a G2/M delay that occurs later in the cell cycle [28].

Since Rts1 is highly conserved, it may also play a role in mechanisms that integrate external signals, cell size, and G1 cyclin transcription in vertebrate cells. The signaling pathways that control G1 cyclin transcription in vertebrate cells are of considerable interest, since deregulation of these pathways contributes to cancer [69].

## Materials and Methods

### Yeast strains, culture conditions, and plasmids

The strains used for this study are listed in Table 2. Cells were grown in yeast extract-peptone-dextrose (YEPD) media supplemented with 40 mg/liter adenine, unless otherwise noted. Full length *CLN2* was expressed from plasmid pSL201-5[*GAL1-CLN2-3XHA URA3*] [49]. 3XHA-tagging of *CLN2* was carried out by digesting plasmid pMT104 with PvuII and integrating at the Cln2 locus using standard yeast transformation techniques [9],[70]. 3XHA-tagging of other genes was carried out as previously described [71].

**Table 2:** Strains used in this study.

Strain	Genotype	Reference or Source
DK186	*MAT*a	Altman *et al.*, 1997 [78]
DK647	*MAT*a *rts1*Δ*::kanMX6*	This study
CC4	*MATa cdc12-6*	Carroll *et al.*, 1998 [34]
DK778	*MATa cdc12-6 rts1*Δ*::kanMX6*	This study
RA19	*MAT*a *gin4*Δ*::LEU2*	Mortensen *et al.*, 2002 [36]
DK655	*MAT*a *gin4*Δ*::LEU2 rts1*Δ*::kanMX6*	This study
AS8	*MAT*α *elm1*Δ*::TRP*	This study
DK682	*MAT*α *elm1*Δ*::TRP rts1*Δ*::kanMX6*	This study
HT160	*MAT*a *cla4*Δ*::HIS3MX6*	Mortensen *et al.*, 2002 [36]
DK738	*MAT*α *cla4*Δ*::HIS3MX6 rts1*Δ*::kanMX6*	This study
AS20	*MAT*a *elm1*Δ*::URA3*	Sreenivasan *et al.*, 2003 [79]
DK847	*MAT*a *elm1*Δ*::LEU2 rts1*Δ*::kanMX6*	This study
DK1027	*MAT*a *GAL1-SWE1::HIS3MX6 CLN2-3XHA::LEU2*	This study
DK1029	*MAT*a *GAL1-SWE1::HIS3MX6 CLN2-3XHA::LEU2 rts1*Δ*::kanMX6*	This study
ZZ41	*MAT*a *CLN2-3XHA::LEU2*	Zimmerman and Kellogg, 2001 [80]
DK751	*MAT*a *CLN2-3XHA::LEU2 rts1*Δ*::kanMX6*	This study
KA61	*MAT*a *cln1*Δ*::TRP cln2*Δ*::LEU2*	This study
DK685	*MAT*α *rts1*Δ*::kanMX6*	This study
KA12	*MATa cln2*Δ*::LEU2*	This study
DK788	*MAT*a *rts1*Δ*::kanMX6 cln2*Δ*::LEU2*	This study
DK547	*MAT*a *cln1*Δ*::TRP GAL1-3XHA-CLN2::HIS3MX6*	This study
DK1158	*MAT*a *cln1*Δ*::TRP GAL1-3XHA-CLN2::HIS3MX6 rts1*Δ*::kanMX6*	This study
DK1300	*MATα cln3*Δ*::HIS3MX6*	This study
DK1309	*MAT*a *rts1*Δ*::kanMX6 cln3*Δ*::HIS3MX6*	This study
ZZ79	*MAT*a *GAL1-3XHA-CLN3*::*HIS3MX6*	This study
DK1419	*MAT*a *GAL1-3XHA-CLN3*::*HIS3MX6 rts1*Δ*::kanMX6*	This study
SH183	*MATa pho85*Δ*::kanMX6*	Egelhofer *et al.*, 2008 [81]
DK853	*MATa pho85*Δ*::kanMX6 rts1*Δ*::HIS3MX6*	This study
DK605	*MATα pcl1*Δ*::natMX6 pcl2::kanMX6*	This study
DK850	*MATα pcl1*Δ*::natMX6 pcl2::kanMX6 rts1*Δ*::HIS3*	This study
DK1561	*MATa GAL1-CDC20::HIS3MX6CLN2-3XHA::LEU2*	This study
DK1549	*MATa GAL1-CDC20:HIS3MX6 CLN2-3XHA::LEU2 rts1*Δ*::kanMX6*	This study
DK1194	*MAT*a pSL201-5[*GAL1-CLN2-3XHA URA3*]	This study
DK1407	*MAT*a ycplac33[CEN/URA3]	This study
DK1233	*MAT*a *rts1*Δ*::HIS3MX6* pSL201-5[*GAL1-Cln2-3XHA URA3*]	This study
DK1408	*MAT*a *rts1*Δ*::kanMX6* ycplac33[CEN/URA]	This study
DK1228	*MAT*a *CLN2-3XHA::LEU2 cdc34-2*	This study
DK1227	*MAT*a *CLN2-3XHA::LEU2 cdc34-2 rts1*Δ*::kanMX6*	This study
DK1304	*MAT*a *CLN2-3XHA::LEU2 whi5*Δ*::kanMX6*	This study
DK1303	*MAT*a *CLN2-3XHA::LEU2 whi5*Δ*::kanMX6 rts1*Δ*::HIS3MX6*	This study
DK456	*MAT*a *whi5*Δ*::kanMX6*	This study
DK1295	*MAT*a *whi5*Δ*::kanMX6 rts1*Δ*::HIS3MX6*	This study
KA46	*MATa bck2*Δ*::kanMX6*	This study
DK818	*MAT*a *rts1*Δ*::HIS3MX6*	This study
DK1320	*MAT*a *rts1*Δ*::HIS3MX6 bck2*Δ*::kanMX6*	This study
DK1307	*MAT*a *CLN2-3XHA::LEU2 rts1*Δ*::HIS3MX6*	This study
DK1329	*MAT*a *CLN2-3XHA::LEU2 bck2*Δ*::kanMX6*	This study
DK1330	*MAT*a *CLN2-3XHA::LEU2 bck2*Δ*::kanMX6 rts1*Δ*::HIS3MX6*	This study
DK1608	*MAT*a *swi4*Δ*::kanMX6*	This study
DK1630	*MAT*a *swi4*Δ*::kanMX6 rts1*Δ*::HIS3MX6*	This study
DK1569	*MAT*α *mbp1*Δ*::kanMX6*	This study
DK1571	*MAT*a *mbp1*Δ*::kanMX6 rts1*Δ*::HIS3MX6*	This study
HT179	*MAT*a *mih1*Δ*::URA3*	Harvey et al., 2003 [82]
DK1415	*MAT*a *SWI6-3XHA::HIS3MX6*	This study
DK1416	*MAT*a *SWI6-3XHA::HIS3MX6 rts1*Δ*::kanMX6*	This study
DK1544	*MAT*a *CLN2-3xHA::LEU2 SWI4-3XHA::HIS3MX6*	This study
DK1543	*MAT*a *CLN2-3xHA::LEU2 SWI4-3XHA::HIS3MX6 rts1*Δ*::kanMX6*	This study
DK1521	*MAT*a *MBP1-3XHA::HIS3MX6*	This study
DK1533	*MAT*a *MBP1-3XHA::HIS3MX6 rts1*Δ*::kanMX6*	This study
DK1417	*MAT*a *STB1-3XHA::HIS3MX6*	This study
DK1418	*MAT*a *STB1-3XHA::HIS3MX6 rts1*Δ*::kanMX6*	This study
DK1498*	*MAT*a *CLN2-3xHA::LEU2*	This study
DK1497*	*MAT*a *CLN2-3xHA::LEU2 rts1*Δ*::kanMX6*	This study
DK889	*MAT*a *CLN2-3XHA::LEU2 gin4*Δ*::HIS3MX6*	This study
DK792	*MAT*a *CLN2-3XHA::LEU2 gin4*Δ*::HIS3MX6 rts1*Δ*::kanMX6*	This study

All strains are isogenic to DK186 and are in the W303 background except as otherwise noted (*leu2-3,112 ura3-1 can1-100 ade2-1 his3-11,15 trp1-1 bar1*Δ* GAL+ ssd1-d2*).

*: BY4741 Strain background (*MAT*a *his3*Δ*1 leu2*Δ*0 met15*Δ*0 ura3*Δ*0 SSD1-v1*).

### Cell cycle time courses

Cells were grown overnight at room temperature on a shaking platform. Cells at an OD_600_ of 0.6 were arrested in G1 by the addition of 0.5 µg/ml α factor for 3.5 hours. Cells were released into a synchronous cell cycle by washing 3× with fresh YEPD pre-warmed to 30°C, and time courses were carried out at 30°C unless otherwise noted. To prevent cells from entering a second cell cycle, α factor was added back at 65 minutes. For metaphase arrest, strains containing *GAL1-CDC20* were first grown overnight in YEP +2% raffinose +2% galactose and then washed into media containing 2% raffinose and allowed to arrest for four hours. Cells were released from metaphase arrest by adding 2% galactose and were then shifted to 30°C. At each time interval, 1.6 ml samples were collected in screw-top tubes. The cells were pelleted, the supernatant was removed, and 250 µl of glass beads were added before flash freezing. To lyse cells, 100 µl of 1× sample buffer (65 mM Tris-HCl (pH 6.8), 3% SDS, 10% glycerol, 50 mM NaF, 50 mM *β*-glycerolphosphate, 5% 2-mercaptoethanol, bromophenol blue) was added. 2 mM PMSF was added to the sample buffer immediately before use from a 100 mM stock made in 100% isopropanol. Cells were lysed by shaking in a Biospec Multibeater-8 at top speed for 2 minutes. The tubes were immediately removed, centrifuged for 1 minute in a microfuge and placed in a boiling water bath for 5 minutes. After boiling, the tubes were again centrifuged for 1 minute and 10 µl was loaded on a gel (5 µl when probing for Nap1). For microscopy, 180 µl samples were collected and fixed by adding 20 µl of 37% formaldehyde for 1 hour. Cells were washed twice in 1× PBS, 0.05% Tween-20, 0.02% sodium azide and imaged by differential interference contrast microscopy. Bud emergence was quantified by counting the number of buds per 200 cells for each sample.

### Western blotting

Polyacrylamide gel electrophoresis was carried out as previously described [72]. Gels were run at 20 mA on the constant current setting. Western blots were transferred for 1 hour at 1 Amp at 4°C in a Hoeffer transfer tank in a buffer containing 20 mM Tris base, 150 mM glycine, and 20% methanol. Blots were probed overnight at 4°C with affinity purified rabbit polyclonal antibodies raised against Clb2, Swe1, Cdc24, Nap1 or HA peptide. Blots were probed with an HRP-conjugated donkey anti-rabbit secondary antibody (GE Healthcare).

For quantitative western blotting, protein was transferred onto Millipore Immobilon-FL membrane. Before transfer, the membrane was first briefly wetted in 100% methanol. A Cy5-conjugated secondary antibody was used (Affinipure goat anti-rabbit, Jackson ImmunoResearch) and images were collected on a Typhoon 9410 variable mode imager. ImageQuant was used to quantify band intensity. Local background was subtracted from each band. An antibody that recognizes the Nap1 protein was used as a loading control. Since Nap1 migrates below Cln2, the western blots could be cut into two pieces to independently probe Cln2 and Nap1 in the same samples. For each lane, the ratio of Cln2/Nap1 signal was determined to normalize for differences in loading. Graphing was done with GraphPad Prism version 4.00 for Mac [73].

### Northern blot analysis

Probes that specifically recognized *CLN1*, *CLN2*, *PCL1*, *RNR1* and *ACT1* RNA were made using a gel-purified PCR product (*CLN1* oligos: AGAATGGTCCTGTAAGAGAAAGT, AGAAACTGATGATGAAGAGGCAT; *CLN2* oligos: TGAACCAATGATCAATGATTACGT, TCAAGTTGGATGCAATTTGCAG; *PCL1* oligos: ACTAGATTGGTCAGATACACCAA, TGGTTACATCTTTTAGCCTTCTTAGA; *RNR1* oligos: ACTTAGGTGTCATCAAGTCATCA, TCTACCACCATGCTTCATGATATCTT; *ACT1* oligos: TCATACCTTCTACAACGAATTGAGA, ACACTTCATGATGGAGTTGTAAGT) and the Megaprime DNA labeling kit (GE Healthcare Amersham). Total yeast RNA was extracted as previously described [74] and blotting was carried out using standard methods [75]. Blots were stripped and re-probed for ACT1 as a loading control. Images were collected on a Typhoon 9410 variable mode imager. ImageQuant was used to quantify band intensity. Local background was subtracted from each band. The amount of *CLN1*, *CLN2*, *PCL1*, and *RNR1* RNA was normalized to the amount of *ACT1* RNA for each lane and used to determine relative expression level. Data from three independent time course experiments was used to determine error bars (standard error of the mean).

### Measurement of the rate of polar growth

Samples of synchronized *GAL1-SWE1* or *GAL1-SWE1 rts1*Δ cells were collected at 30 minute intervals following release from a G1 arrest into galactose-containing media. Cells were imaged using differential interference contrast microscopy and ImageJ 1.37v software was used to measure bud length and bud width for 100 cells for each sample [76]. The extent of polar growth was measured as the ratio of length/width, and was plotted for each time point.

### Determination of the half-life of the Cln2 protein

The half-life of the Cln2 protein was determined as previously described with the following modifications [49]. Cells were grown overnight in YEP media containing 2% glycerol/ethanol. *GAL1-CLN2* transcription was induced by washing cells into YEP media containing 2% galactose for 1 hour. Expression of *CLN2* was then repressed by washing cells into YEPD media. Quantitative Western blotting was carried out to quantify Cln2 levels over time. Non-linear regression curve fitting for one phase exponential decay was carried out using GraphPad Prism version 4.00 for Mac [73].

### Measurement of cell size

Cultures of cells were grown in triplicate overnight at 25°C in either YEP +2% dextrose or YEP +2% glycerol and 2% ethanol. A 1 ml sample of log phase (OD_600_ = 0.60) culture was fixed with 1/10 volume 37% formaldehyde for 1 hour, then washed twice with 1× PBS+0.04% sodium azide +0.02% Tween 20. Cell size was measured using a Channelizer Z2 Coulter counter as previously described [77]. 150 µl of fixed culture were diluted in 20 ml of Isoton II and sonicated for 20 seconds prior to Coulter counter analysis. Each plot is the average of 4 independent experiments in which 3 independent samples were analyzed.

## Supporting Information

Figure S1 *rts1*Δ results in delayed G1 events. (A) The Cdc24 protein undergoes normal dephosphorylation during release from α factor arrest in *rts1*Δ cells. Western blotting was carried out with an anti-Cdc24 antibody. Changes in Cdc24 phosphorylation were detected as a shift in electrophoretic mobility. (B) Accumulation of the Cln2 protein is delayed during a synchronized cell cycle in *rts1*Δ cells in the S288C (*SSD1*-v1) strain background. Wild type and *rts1*Δ cells were released from an α factor arrest into pre-warmed media at 30°C. Levels of Cln2-3XHA were monitored by Western blotting. (C) *CLN1* and *PCL1* RNA accumulation were delayed and reduced in *rts1*Δ cells. Wild type and *rts1*Δ cells were released from an α factor arrest into pre-warmed media at 30°C and samples were collected at 10 minute intervals during the cell cycle. Levels of *CLN1* and *PCL1* mRNA were monitored by Northern blotting.
(0.94 MB TIF)

Figure S2 Cln2 accumulation is delayed and reduced in *gin4*Δ *rts1*Δ cells. *gin4*Δ and *rts1*Δ *gin4*Δ cells were released from an α factor arrest into pre-warmed media at 30°C. Levels of Cln2-3XHA and Clb2 were monitored by Western blotting.
(0.66 MB TIF)
