# Supplementary figures and images for: The Rts1 Regulatory Subunit of Protein Phosphatase 2A Is Required for Control of G1 Cyclin Transcription and Nutrient Modulation of Cell Size
Source: PLoS Genet. 2009 Nov 13;5(11):e1000727. doi: 10.1371/journal.pgen.1000727 (PMC2770260; doi:10.1371/journal.pgen.1000727)

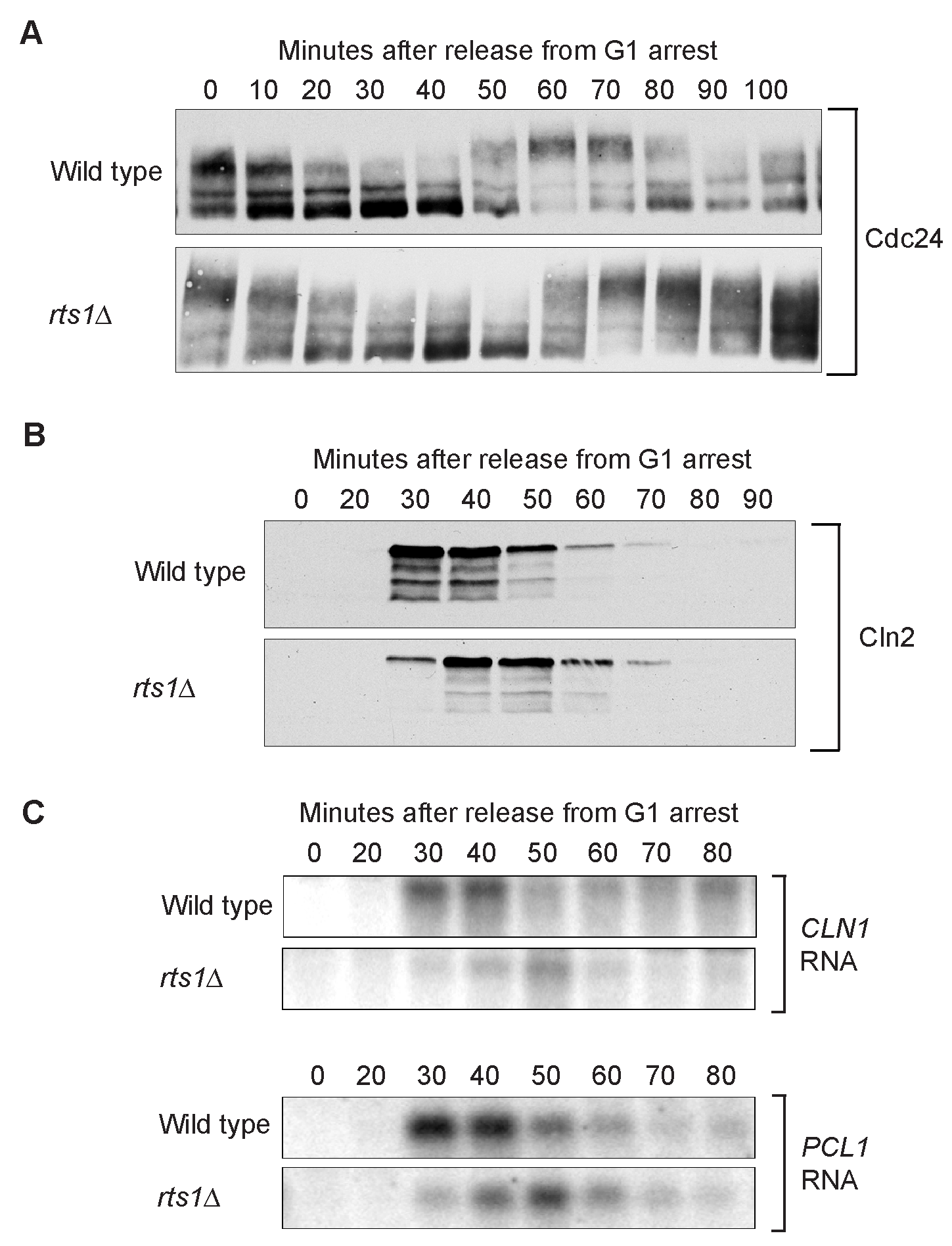

Supplement: Figure S1 — rts1Δ results in delayed G1 events. (A) The Cdc24 protein undergoes normal dephosphorylation during release from α factor arrest in rts1Δ cells. Western blotting was carried out with an anti-Cdc24 antibody. Changes in Cdc24 phosphorylation were detected as a shift in electrophoretic mobility. (B) Accumulation of the Cln2 protein is delayed during a synchronized cell cycle in rts1Δ cells in the S288C (SSD1-v1) strain background. Wild type and rts1Δ cells were released from an α factor arrest into pre-warmed media at 30°C. Levels of Cln2-3XHA were monitored by Western blotting. (C) CLN1 and PCL1 RNA accumulation were delayed and reduced in rts1Δ cells. Wild type and rts1Δ cells were released from an α factor arrest into pre-warmed media at 30°C and samples were collected at 10 minute intervals during the cell cycle. Levels of CLN1 and PCL1 mRNA were monitored by Northern blotting. (0.94 MB TIF) [file pgen.1000727.s001.tif]

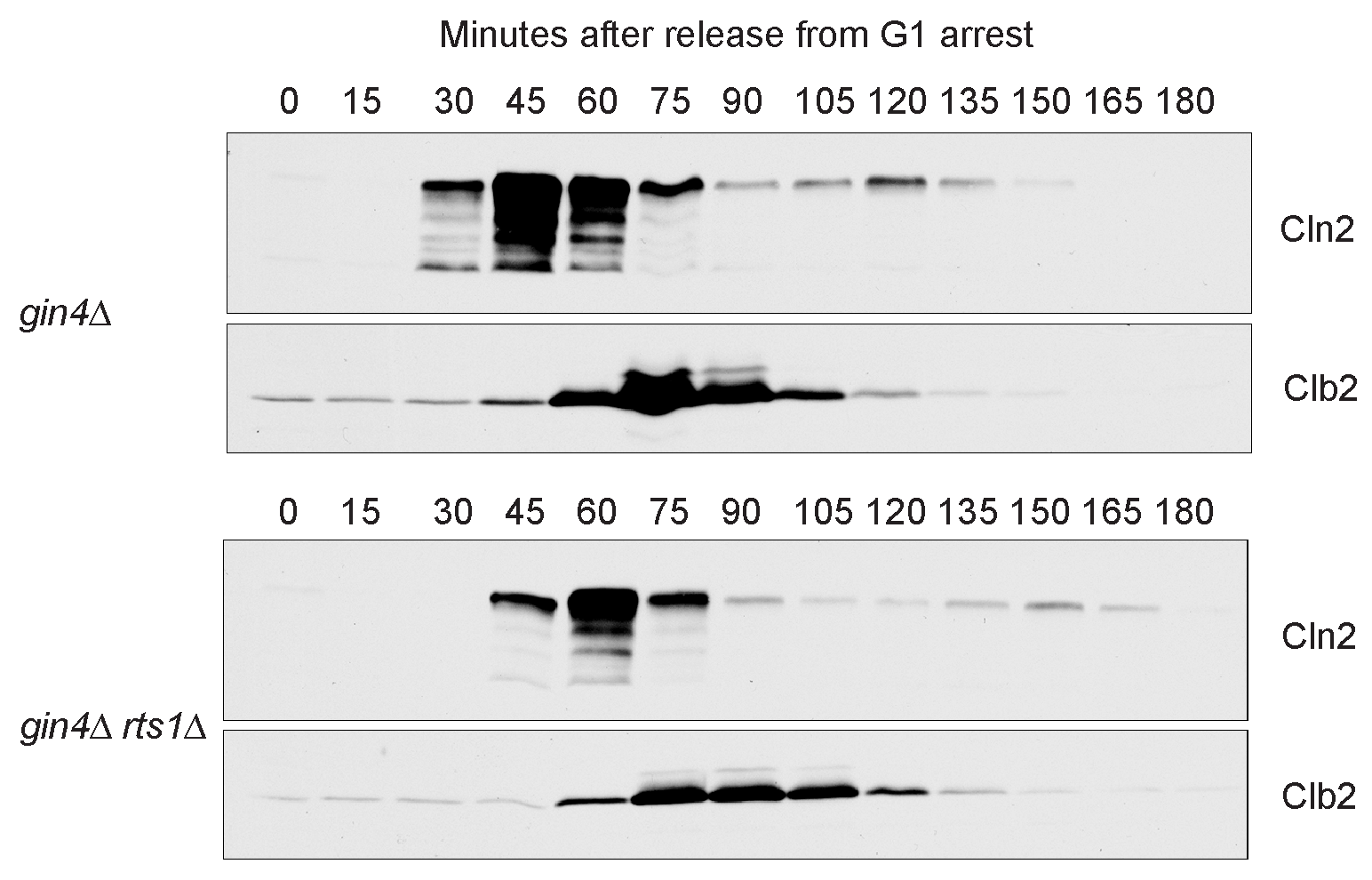

Supplement: Figure S2 — Cln2 accumulation is delayed and reduced in gin4Δ rts1Δ cells. gin4Δ and rts1Δ gin4Δ cells were released from an α factor arrest into pre-warmed media at 30°C. Levels of Cln2-3XHA and Clb2 were monitored by Western blotting. (0.66 MB TIF) [file pgen.1000727.s002.tif]
